# Supplementary material for: Molecular Modelling of Ionic Liquids: Situations When Charge Scaling Seems Insufficient
Source: Molecules. 2023 Jan 13;28(2):800. doi: 10.3390/molecules28020800 (PMC9865557; doi:10.3390/molecules28020800)
Supplement: Supplementary file 1 [file molecules-28-00800-s001.zip › molecules-2162228-supplementary.pdf]

Supporting Information:

Molecular Modelling of Ionic Liquids: Situations When Charge Scaling Seems Insufficient

Zhaoxi Sun[1,*], Lei Zheng[2,3], Zuo-yuan Zhang[4], Yalong Cong[5], Mao Wang[6], Xiaohui Wang[1,7,*], Jingjing Yang[8], Zhirong Liu[1], Zhe Huai[9,*]

[1]*College of Chemistry and Molecular Engineering, Peking University, Beijing 100871, China*

[2]*NYU-ECNU Center for Computational Chemistry at NYU Shanghai, Shanghai 200062, China*

[3]*Department of Chemistry, New York University, New York, NY10003, USA*

[4]*College of Physical Science and Technology, Yangzhou University, Yangzhou 225009, China*

[5]*School of Chemistry and Molecular Engineering, East China Normal University, Shanghai 200062, China*

[6]*NCS Testing Technology Co., Ltd., No. 13, Gaoliangqiao Xiejie, Beijing 100081, China*

[7]*Beijing Leto Laboratories Co., Ltd., Beijing 100083, China*

[8]*School of Environmental Science and Engineering, Suzhou University of Science and Technology, Suzhou 215009, China*

[9]*XtalPi - AI Research Center, 7F, Tower A, Dongsheng Building, No.8, Zhongguancun East Road, Beijing 100083, China*

Fig. S1. Comparison between the experimental and calculated mass densities along the temperature ladder. The results obtained with the original GAFF2 vdW parameters are systematically larger than experiment in the whole temperature range, while the 1.03-σ-scaled estimates are significantly improved and close to the reference data. For the [QUIN8][NTF] ionic solvent, the 1.03-scaled parameter set experiences a performance loss at elevated temperatures, and the best vdW scaling factor lies between 1.03 and 1.05. This observation is in agreement with the 298 K scan shown in Fig. 4b, where the best vdW scaling factor for this solvent is found to be slightly larger than 1.03. As we are using the same vdW scaling factor for the two ILs, the 1.03 option is indeed achieving better performance than 1.05 or the normal 100% GAFF2 that performs rather poorly for [QUIN6][NTF].

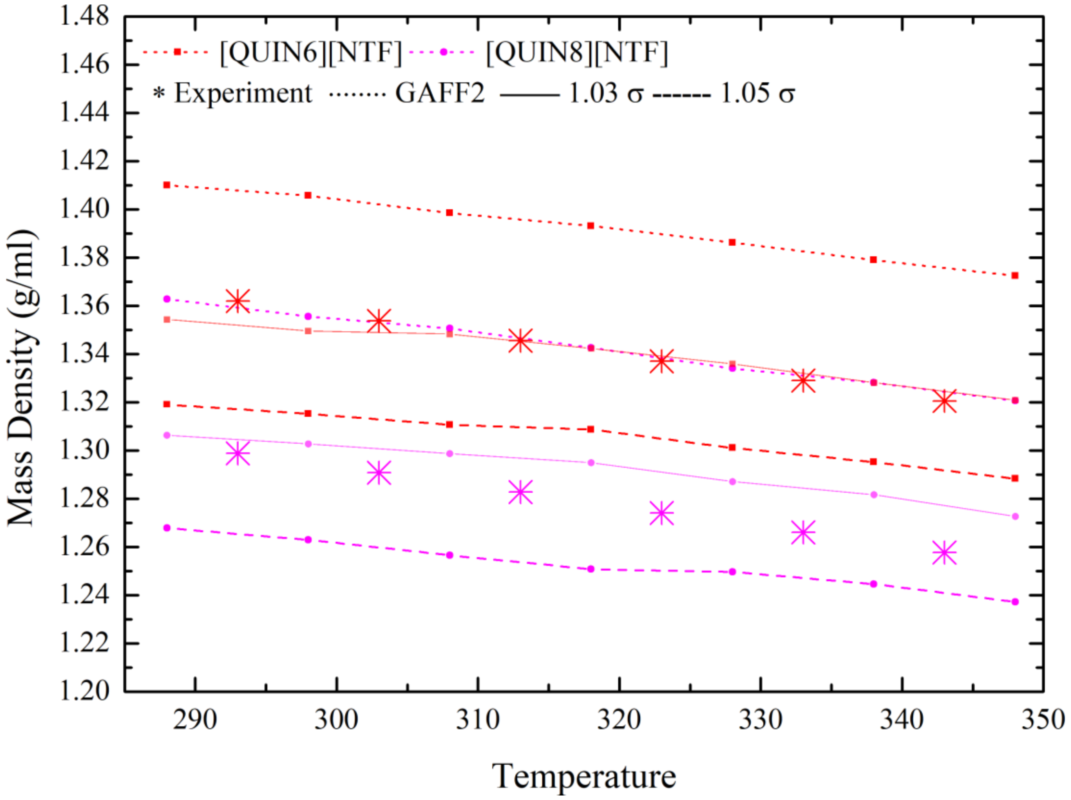

Table S1. Hydration free energies, solvation free energies in [QUIN6][NTF], and the water-[QUIN6][NTF] transfer free energies in kcal/mol. GAFF2, 103% σ and 105% σ represent the free energy estimates obtained with the normal GAFF2 vdW parameters, 103%-scaled vdW radius, and 105%-scaled vdW radius. SD represents the standard error of the computed free energy estimate, which is obtained from numerical bootstrap resampling. MSE, MAE, RMSE, τ, PI and Pearson *r* serve as quality measurements.

Solute	Solvation in Water (kcal/mol)			Solvation in Ionic Solvent (kcal/mol)							Water-Ionic-Liquids Transfer (kcal/mol)						
	ΔG_{exp}	ΔG_{calc}	SD	ΔG_{exp}	GAFF2	SD	1.03 σ	SD	1.05 σ	SD	ΔG_{exp}	GAFF2	SD	1.03 σ	SD	1.05 σ	SD
1,4-dioxane	-5.05	-4.05	0.06	-4.55	-4.87	0.17	-4.43	0.21	-5.16	0.60	-0.50	0.82	0.18	0.38	0.22	1.11	0.60
1-butanol	-4.72	-3.83	0.10	-4.75	-3.98	0.31	-4.11	0.18	-3.92	0.52	0.03	0.15	0.33	0.28	0.21	0.09	0.53
1-hexene	1.68	1.34	0.04	-2.49	-2.23	0.44	-1.59	0.24	-2.45	0.22	4.17	3.57	0.44	2.93	0.24	3.79	0.23
1-hexyne	0.29	-0.48	0.04	-3.17	-4.24	0.38	-3.20	0.27	-4.78	0.53	3.46	3.76	0.38	2.72	0.27	4.30	0.53
1-nitropropane	-3.34	-4.38	0.03	-5.31	-5.26	0.19	-5.87	0.50	-4.38	0.88	1.97	0.88	0.20	1.49	0.50	0.00	0.88
1-propanol	-4.83	-4.06	0.10	-4.16	-3.23	0.16	-4.07	0.43	-3.79	0.67	-0.67	-0.83	0.19	0.01	0.45	-0.27	0.68
2,2,4-trimethylpentane	2.85	1.79	0.03	-2.71	-3.02	0.73	-1.59	0.27	-1.32	0.94	5.56	4.81	0.73	3.38	0.27	3.11	0.94
2-butanone	-3.64	-3.25	0.03	-4.17	-4.11	0.37	-3.71	0.47	-2.81	0.32	0.53	0.86	0.37	0.46	0.47	-0.44	0.33
2-methyl-1-propanol	-4.52	-4.15	0.17	-4.47	-3.76	0.23	-3.57	0.39	-3.75	0.43	-0.05	-0.39	0.28	-0.58	0.42	-0.40	0.46
2-pentanone	-3.53	-3.59	0.03	-4.64	-4.73	0.23	-5.05	0.59	-5.57	0.36	1.11	1.14	0.24	1.46	0.59	1.98	0.36
2-propanol	-4.76	-3.97	0.11	-3.77	-4.52	0.25	-4.29	0.47	-4.04	0.45	-0.99	0.55	0.27	0.32	0.48	0.07	0.46
3-methylpentane	2.51	2.18	0.04	-2.11	-1.94	0.34	-1.14	0.65	-3.57	1.10	4.62	4.12	0.34	3.32	0.65	5.75	1.10
acetone	-3.85	-3.59	0.04	-3.70	-3.62	0.22	-4.56	0.38	-4.05	0.52	-0.15	0.03	0.22	0.97	0.38	0.46	0.52
acetonitrile	-3.89	-3.92	0.02	-4.08	-4.15	0.24	-4.24	0.27	-3.73	0.40	0.19	0.23	0.24	0.32	0.27	-0.19	0.40
benzene	-0.87	-1.36	0.04	-4.04	-3.72	0.28	-4.83	0.67	-3.19	0.59	3.17	2.36	0.29	3.47	0.68	1.83	0.59
chloroform	-1.07	0.09	0.03	-3.67	-3.92	0.28	-3.70	0.42	-2.94	0.22	2.60	4.01	0.28	3.79	0.42	3.03	0.23
cyclohexane	1.23	1.04	0.04	-2.70	-1.30	0.29	-2.92	0.35	-2.60	0.84	3.93	2.34	0.29	3.96	0.36	3.64	0.85
decane	3.22	2.83	0.04	-4.47	-3.52	0.52	-2.25	0.60	-4.65	1.51	7.69	6.35	0.52	5.08	0.60	7.48	1.51
dichloromethane	-1.36	-0.43	0.03	-3.09	-2.60	0.16	-3.66	0.19	-2.96	0.27	1.73	2.17	0.17	3.23	0.20	2.53	0.27
diethyl ether	-1.76	-0.05	0.06	-2.42	-2.02	0.22	-1.89	0.33	-2.56	0.44	0.66	1.97	0.23	1.84	0.33	2.51	0.45
diisopropyl ether	-0.53	-1.54	0.06	-2.79	-3.74	0.66	-2.83	0.79	-1.52	0.70	2.26	2.20	0.66	1.29	0.79	-0.02	0.71
ethanol	-5.01	-4.18	0.09	-3.61	-3.50	0.24	-3.99	0.39	-2.56	0.32	-1.40	-0.68	0.26	-0.19	0.40	-1.62	0.33
ethyl acetate	-3.10	-3.77	0.07	-3.90	-3.84	0.43	-3.29	0.29	-4.27	0.23	0.80	0.07	0.44	-0.48	0.30	0.50	0.24
ethylbenzene	-0.80	-1.14	0.04	-5.06	-5.19	0.59	-3.92	0.57	-4.52	0.91	4.26	4.05	0.59	2.78	0.57	3.38	0.91
heptane	2.62	2.31	0.05	-2.76	-1.67	0.22	-0.71	0.19	-1.34	0.62	5.38	3.98	0.23	3.02	0.20	3.65	0.62
hexane	2.49	2.28	0.04	-2.19	-1.95	0.27	-1.41	0.36	-1.67	0.47	4.68	4.23	0.27	3.69	0.36	3.95	0.47
methanol	-5.11	-3.97	0.08	-3.26	-2.69	0.13	-2.72	0.27	-2.97	0.40	-1.85	-1.28	0.16	-1.25	0.28	-1.00	0.40
methylcyclohexane	1.71	1.14	0.04	-3.05	-2.97	0.27	-3.01	0.46	-2.81	0.61	4.76	4.11	0.27	4.15	0.46	3.95	0.61
methylcyclopentane	1.60	1.23	0.04	-2.53	-4.10	0.70	-3.13	0.74	-2.78	0.50	4.13	5.33	0.70	4.36	0.74	4.01	0.50
m-xylene	-0.84	-0.91	0.03	-5.17	-6.15	0.38	-6.12	0.38	-3.98	1.09	4.33	5.24	0.38	5.21	0.38	3.07	1.09
nitromethane	-3.95	-4.91	0.02	-4.55	-5.12	0.19	-5.67	0.41	-5.35	0.20	0.60	0.21	0.19	0.76	0.41	0.44	0.20
nonane	3.04	2.39	0.05	-3.88	-3.82	0.56	-4.11	0.47	-2.95	0.71	6.92	6.21	0.56	6.50	0.47	5.34	0.71
octane	2.89	2.46	0.05	-3.40	-2.94	0.42	-2.04	0.36	-2.47	0.69	6.29	5.40	0.43	4.50	0.36	4.93	0.69
o-xylene	-0.90	-0.92	0.03	-5.41	-3.54	0.34	-3.58	0.59	-3.48	0.37	4.51	2.62	0.34	2.66	0.59	2.56	0.37
p-xylene	-0.81	-0.76	0.05	-5.15	-5.17	0.19	-2.98	0.56	-1.71	0.30	4.34	4.41	0.19	2.22	0.56	0.95	0.31
pyridine	-4.70	-3.00	0.04	-5.09	-4.25	0.31	-4.42	0.51	-4.57	0.44	0.39	1.25	0.31	1.42	0.51	1.57	0.44

tetrahydrofuran	-3.47	-2.26	0.04	-3.77	-4.00	0.64	-3.70	0.24	-4.56	0.44	0.30	1.74	0.64	1.44	0.24	2.30	0.44
thiophene	-1.42	-1.55	0.04	-4.19	-4.20	0.25	-4.06	0.42	-4.46	0.61	2.77	2.65	0.25	2.51	0.43	2.91	0.61
toluene	-0.89	-1.04	0.03	-4.61	-4.55	0.57	-3.84	0.61	-2.34	0.25	3.72	3.51	0.57	2.80	0.61	1.30	0.25
triethylamine	-3.02	1.00	0.06	-3.07	-2.86	0.24	-1.67	0.29	-0.42	0.28	0.05	3.86	0.25	2.67	0.30	1.42	0.29
RMSE		0.98			0.67		0.93		1.14			1.05		1.24		1.32	
MSE		-0.17			-0.12		-0.35		-0.48			-0.04		0.19		0.31	
MAE		0.70			0.49		0.72		0.88			0.79		1.02		1.07	
τ		0.74			0.54		0.47		0.35			0.74		0.68		0.64	
PI		0.93			0.65		0.62		0.45			0.89		0.87		0.85	
Pearson *r*		0.94			0.79		0.73		0.55			0.90		0.88		0.85	

Table S2. Hydration free energies, solvation free energies in [QUIN8][NTF], and the water-[QUIN8][NTF] transfer free energies in kcal/mol. GAFF2, 103% σ, and 105% σ represent the free energy estimates obtained with the normal GAFF2 vdW parameters, 103%-scaled vdW radius, and 105%-scaled vdW radius. SD represents the standard error of the computed free energy estimate, which is obtained from numerical bootstrap resampling. MSE, MAE, RMSE, τ, PI and Pearson *r* serve as quality measurements.

Solute	Solvation in Water (kcal/mol)			Solvation in Ionic Solvent (kcal/mol)							Water-Ionic-Liquids Transfer (kcal/mol)						
	ΔG_{exp}	ΔG_{calc}	SD	ΔG_{exp}	GAFF2	SD	1.03 σ	SD	1.05 σ	SD	ΔG_{exp}	GAFF2	SD	1.03 σ	SD	1.05 σ	SD
1,4-dioxane	-5.05	-4.05	0.06	-4.57	-5.40	0.69	-5.52	0.35	-6.92	0.75	-0.48	1.35	0.69	1.47	0.36	2.87	0.75
1-butanol	-4.72	-3.83	0.10	-4.76	-3.36	0.23	-5.71	0.51	-5.48	0.38	0.04	-0.47	0.25	1.88	0.52	1.65	0.39
1-hexene	1.68	1.34	0.04	-2.67	-2.10	0.27	-2.38	0.46	-3.55	1.04	4.35	3.44	0.27	3.72	0.46	4.89	1.04
1-nitropropane	-3.34	-4.38	0.03	-5.34	-6.37	0.77	-6.66	0.80	-8.35	0.34	2.00	1.99	0.77	2.28	0.80	3.97	0.35
1-propanol	-4.83	-4.06	0.10	-4.16	-3.42	0.22	-4.83	0.46	-4.43	0.26	-0.67	-0.64	0.24	0.77	0.47	0.37	0.28
2,2,4-trimethylpentane	2.85	1.79	0.03	-3.03	-2.99	0.58	-2.82	0.58	-3.45	1.22	5.88	4.78	0.58	4.61	0.58	5.24	1.22
2-butanone	-3.64	-3.25	0.03	-4.19	-3.44	0.30	-4.90	0.86	-4.93	0.30	0.55	0.19	0.30	1.65	0.86	1.68	0.30
2-methyl-1-propanol	-4.52	-4.15	0.17	-4.48	-4.26	0.23	-4.09	0.23	-5.92	0.28	-0.04	0.11	0.29	-0.06	0.29	1.77	0.33
2-pentanone	-3.53	-3.59	0.03	-4.68	-4.32	0.34	-5.66	0.99	-5.27	0.37	1.15	0.73	0.34	2.07	0.99	1.68	0.37
2-propanol	-4.76	-3.97	0.11	-3.76	-3.30	0.18	-3.69	0.60	-3.71	0.27	-1.00	-0.67	0.21	-0.28	0.61	-0.26	0.30
3-methylpentane	2.51	2.18	0.04	-2.42	-2.24	0.49	-3.08	0.65	-5.32	0.36	4.93	4.42	0.49	5.26	0.65	7.50	0.36
acetone	-3.85	-3.59	0.04	-3.70	-3.42	0.12	-4.02	0.62	-3.76	0.43	-0.15	-0.17	0.12	0.43	0.62	0.17	0.43
acetonitrile	-3.89	-3.92	0.02	-4.05	-4.04	0.21	-4.04	0.12	-5.73	0.35	0.16	0.12	0.21	0.12	0.12	1.81	0.35
benzene	-0.87	-1.36	0.04	-4.07	-3.60	0.48	-4.38	0.29	-5.24	0.63	3.20	2.24	0.48	3.02	0.29	3.88	0.63
chloroform	-1.07	0.09	0.03	-3.72	-3.21	0.21	-3.44	0.21	-5.40	0.60	2.65	3.30	0.22	3.53	0.21	5.49	0.60
cyclohexane	1.23	1.04	0.04	-2.99	-2.82	0.52	-3.12	0.24	-5.15	0.75	4.22	3.86	0.52	4.16	0.24	6.19	0.75
decane	3.22	2.83	0.04	-4.73	-5.05	0.60	-3.71	0.56	-1.80	0.88	7.95	7.88	0.60	6.54	0.56	4.63	0.88
dichloromethane	-1.36	-0.43	0.03	-3.13	-3.31	0.23	-3.19	0.24	-3.53	0.21	1.77	2.88	0.23	2.76	0.24	3.10	0.21
diethyl ether	-1.76	-0.05	0.06	-2.53	-2.13	0.23	-3.06	0.50	-5.14	0.26	0.77	2.08	0.24	3.01	0.51	5.09	0.27
diisopropyl ether	-0.53	-1.54	0.06	-2.91	-3.58	0.53	-3.42	0.47	-3.65	1.33	2.38	2.04	0.53	1.88	0.47	2.11	1.33
ethanol	-5.01	-4.18	0.09	-3.59	-3.20	0.28	-3.34	0.22	-3.90	0.22	-1.42	-0.98	0.30	-0.84	0.24	-0.28	0.24
ethyl acetate	-3.10	-3.77	0.07	-3.94	-3.61	0.19	-5.00	0.38	-6.70	1.07	0.84	-0.16	0.21	1.23	0.39	2.93	1.07
ethylbenzene	-0.80	-1.14	0.04	-5.13	-5.24	0.51	-5.28	0.38	-6.63	0.41	4.33	4.10	0.52	4.14	0.38	5.49	0.41
heptane	2.62	2.31	0.05	-3.07	-3.69	0.37	-3.80	0.29	-3.27	0.34	5.69	6.00	0.37	6.11	0.29	5.58	0.34
hexane	2.49	2.28	0.04	-2.51	-2.71	0.74	-3.85	0.42	-4.17	0.97	5.00	4.99	0.74	6.13	0.42	6.45	0.97
methanol	-5.11	-3.97	0.08	-3.20	-2.63	0.20	-2.35	0.16	-1.93	0.13	-1.91	-1.34	0.21	-1.62	0.18	-2.04	0.15
methylcyclohexane	1.71	1.14	0.04	-3.31	-3.25	0.42	-5.53	0.63	-6.77	0.57	5.02	4.39	0.42	6.67	0.63	7.91	0.57
methylcyclopentane	1.60	1.23	0.04	-2.78	-2.67	0.42	-3.17	0.29	-5.22	0.77	4.38	3.90	0.42	4.40	0.29	6.45	0.77
m-xylene	-0.84	-0.91	0.03	-5.24	-5.07	0.90	-4.67	0.32	-6.25	0.38	4.40	4.16	0.90	3.76	0.32	5.34	0.38
nitromethane	-3.95	-4.91	0.02	-4.51	-5.47	0.22	-5.64	0.31	-5.64	0.22	0.56	0.56	0.22	0.73	0.31	0.73	0.22
nonane	3.04	2.39	0.05	-4.17	-3.76	0.43	-3.55	0.28	-1.62	0.57	7.21	6.15	0.44	5.94	0.29	4.01	0.57
octane	2.89	2.46	0.05	-3.63	-3.63	0.38	-3.73	0.49	-3.79	0.58	6.52	6.09	0.38	6.19	0.50	6.25	0.59
o-xylene	-0.90	-0.92	0.03	-5.44	-4.34	0.47	-5.21	0.36	-4.72	0.33	4.54	3.42	0.47	4.29	0.36	3.80	0.33
p-xylene	-0.81	-0.76	0.05	-5.20	-4.96	0.49	-4.48	0.27	-6.22	0.36	4.39	4.20	0.49	3.72	0.28	5.46	0.37
pyridine	-4.70	-3.00	0.04	-5.09	-5.06	0.78	-4.71	0.39	-5.94	0.45	0.39	2.06	0.78	1.71	0.40	2.94	0.45
tetrahydrofuran	-3.47	-2.26	0.04	-3.82	-3.40	0.17	-5.97	0.76	-5.15	0.33	0.35	1.14	0.18	3.71	0.76	2.89	0.34

thiophene	-1.42	-1.55	0.04	-4.22	-3.76	0.15	-4.88	0.39	-6.03	0.38	2.80	2.21	0.16	3.33	0.40	4.48	0.38
toluene	-0.89	-1.04	0.03	-4.66	-4.42	0.20	-4.31	0.51	-6.98	1.11	3.77	3.38	0.20	3.27	0.51	5.94	1.11
triethylamine	-3.02	1.00	0.06	-3.26	-2.60	0.35	-5.18	0.78	-3.94	0.47	0.24	3.60	0.35	6.18	0.79	4.94	0.48
RMSE		0.98			0.54		0.86		1.68			0.89		1.43		2.00	
MSE		-0.19			-0.18		0.35		1.00			-0.01		-0.54		-1.19	
MAE		0.69			0.43		0.67		1.38			0.63		0.95		1.63	
τ		0.73			0.68		0.51		0.35			0.82		0.74		0.62	
PI		0.93			0.88		0.70		0.45			0.93		0.87		0.83	
Pearson *r*		0.94			0.86		0.68		0.44			0.94		0.86		0.79	
